# Von Mises stresses on Mushroom-loop archwires for incisor retraction: a numerical study

**DOI:** 10.1590/2177-6709.25.4.044-050.oar

**Published:** 2020

**Authors:** Marcelo do Amaral Ferreira, Fábio Rodrigo Mandello Rodrigues, Marco Antônio Luersen, Paulo César Borges, Ravindra Nanda, Marcio Rodrigues de Almeida

**Affiliations:** 1 Private Practice (Curitiba/PR, Brazil).; 2 Universidade Tecnológica Federal do Paraná, Departamento Acadêmico de Mecânica (Curitiba and Pato Branco/PR, Brazil).; 3 University of Connecticut, Department of Craniofacial Sciences (Farmington/CT, USA).; 4 Universidade Norte do Paraná, Departamento de Ortodontia (Londrina/PR, Brazil).

**Keywords:** Mushroom archwires, Finite Element Method, Titanium-molybdenum alloys

## Abstract

**Objective:**

To perform a numerical simulation using FEM to study the von Mises stresses on Mushroom archwires.

**Methods:**

Mushroom archwires made of titanium-molybdenum alloy with 0.017 x 0.025-in cross-section were used in this study. A YS of 1240 MPa and a Young’s modulus of 69 GPa were adopted. The archwire was modeled in Autodesk Inventor software and its behavior was simulated using the finite element code Ansys Workbench (Swanson Analysis Systems, Houston, Pennsylvania, USA). A large displacement simulation was used for non-linear analysis. The archwires were deformed in their extremities with 0° and 45°, and activated by their vertical extremities separated at 4.0 or 5.0 mm.

**Results:**

Tensions revealed a maximum of 1158 MPa at the whole part of the loop at 5.0mm of activation, except in a very small area situated at the top of the loop, in which a maximum of 1324 Mpa was found.

**Conclusions:**

Mushroom loops are capable to produce tension levels in an elastic range and could be safely activated up to 5.0mm.

## RESUMO

**Objetivo:**

Realizar uma simulação numérica, por meio do Método dos Elementos Finitos (MEF), para obter as tensões de von Mises em arcos Mushroom.

**Métodos:**

Foram usados arcos com geometria Mushroom de titânio-molibdênio, com secção transversal 0,017” x 0,025”. Adotou-se valores de tensão de escoamento (σesc) de 1.240 MPa e módulo de elasticidade (E) de 69 GPa. O arco foi modelado por meio do *software* Autodesk Inventor, e seu desempenho foi simulado utilizando-se o *software* de elementos finitos Ansys Workbench (Swanson Analysis System, Houston, Pennsylvania, EUA). Para a simulação, foi considerada a análise para grandes deslocamentos. O arco foi conformado em suas extremidades considerando-se planos de 0° e 45°, pré-ativado em 2,5mm e ativado por meio de suas extremidades verticais, separadas 4,0 mm ou 5,0 mm.

**Resultados:**

As tensões revelaram um valor máximo de 1.158 MPa na maior parte da alça, aos 5,0 mm de ativação, com exceção de uma área muito pequena, com valor de 1.324 MPa, situada no topo da alça.

**Conclusões:**

Os arcos Mushroom são capazes de produzir níveis de tensão situados dentro da região elástica e poderiam ser ativados com segurança até os 5,0 mm de ativação.

## INTRODUCTION

Malocclusion treatment requires a treatment plan considering a 3-D approach, since dental arches can have an elliptic, hyperbolic, parabolic, U-shaped or a V-shaped form. Also, in dental movement, teeth must be considered in a 3-D spatial position. The 3D Finite Element Analysis (FEM) has been widely used for the analysis of complex structures under different loads and conditions,^1^ ^-^ ^2^ because one of the most important parts of numerical analysis is to minimize loss of performance of a structure. Also, it allows to create, develop and test the mechanical behavior of many appliances not only in medicine, but also in dentistry, engineering and other biomedical fields of interest, as structural analysis, heat transfer, mass transport, fluid flow and electromagnetic force.^1^ Once teeth are attached in the periodontal ligament, a complex force system is created during orthodontic treatment. Many papers deal with periodontium modeling, i.e., gingiva, periodontal ligament, alveolar bone and cementum, to study teeth movement in all space positions, but modeling is complex due to anatomical differences among patients.^1^ ^-^ ^2^ Orthodontic appliances should be developed to move teeth with a desired force system (Fx, Fy and Mz) capable to produce controlled teeth tipping, intrusion or extrusion, during movement^3^. In orthodontics, closing loops are used to retract teeth in cases where spaces need to be closed to obtain a stable occlusion, considering that many spaces are due to therapeutic extraction cases in dental protrusion treatment. Closing loops have been studied from the first design from Bull,^4^ who developed a canine retraction spring made with stainless steel. Later, Burstone^5^ developed T-loop closing loops made with titanium-molybdenum alloy. Among the different geometries, T-loops were the most studied at moment. T-loops have been verified in holographic studies, also experimentally, numerically and clinically along the years, regarding gable bends effects, loop position, cross-section, relaxation stress,^6^ and the behavior of the force system.^3^ ^,^ ^5^ Closing loops archwires have been studied experimentally^2^ ^,^ ^3^ and numerically^3^ to obtain tension levels and the force system, i.e., three-dimension forces (Fx, Fy and Fz), rotational tendency (Mx, My and Mz) and consequently the M/F ratios (M/F ratios are relevant to know the tooth movement tendency). From the classic studies on closing loops to the present date, many designs have been created and some of them had their tension levels evaluated through von Mises yield criterion. The von Mises yield criterion through FEM predicts the stress (yielding of materials) on ductile materials over a material under complex loading (multiaxial loading conditions). Some years ago, a modified T-loop called Mushroom loop archwires (ML archwires) was developed^7^ to retract incisors with controlled torque and anchorage control, since this archwire avoids posterior teeth to move forward, resulting in anchorage loss. If molars move mesially, the extraction spaces may be lost and dental protrusion may not be perfectly corrected.^7^ ^-^ ^12^

No study at this time verified the tension levels on Mushroom archwires geometry, but only clinical studies were performed showing good results.^7^ ^,^ ^9^ Thus, the present study aims to study using FEM the von Mises stresses over a 3-D Mushroom loop design after activation.

## MATERIALS AND METHODS

Tridimensional model

**Figure 1:**
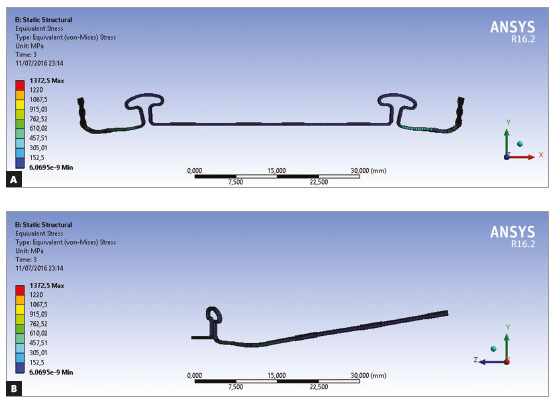
A) Mushroom archwire isometric frontal view. B) Mushroom archwire isometric sagittal view.

**Figure 2:**
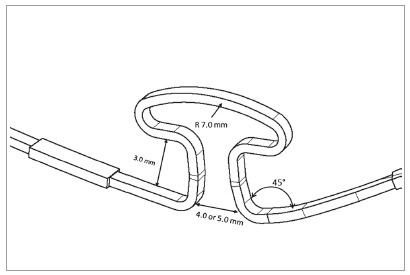
Mushroom angular and linear dimensions.

The archwire was modeled in AutoDesk Inventor software and its behavior was simulated using the finite element code Ansys Workbench (Swanson Analysis Systems, Houston, Pennsylvania, USA). The FEM consist in splitting the body into sub-regions, the FEs. The equations pertaining to each element are joined to preserve continuity and obtain an equation that represents the entire body. In the static analysis of stress-strain, the equation [K] x {u} = {F} represents the body to be analyzed. The ‘K’ is the stiffness matrix, ‘u’ is the nodal displacement vector, and ‘F’ is the nodal force vector. After finding the nodal displacements {u} through the solution of the algebraic system shown in equation above, the stresses and efforts on the body may be evaluated. Thus, the matrix [K] depends on the vector {u}, characterizing a non-linear system of equations due to large displacements characteristics. The activation was performed in increments of 1.0 mm in the horizontal direction up to 5.0 mm, and considered maximum when, at any point, the archwire material reached its YS (Yield Strength) limit and, consequently, suffered permanent deformation, and the simulation process was terminated. Figures 1A and 1B show the isometric view. Figure 2 depicts the loop dimensions with angular and linear details. The archwire is characterized by 0.432 x 0.635 mm (0.017 x 0.025-in) cross-section and made by titanium-molybdenum alloy with a Modulus of Elasticity (E) of 69 GPa (10 x 10^6^psi) and a YS (σe) equal to 1240 MPa (180 x 10^3^psi). 

### Tension analysis

Stress analysis is performed to verity if a ductile material is working in the elastic regime, and serves as a parameter to define the maximal admissible activation, since plastic deformation should be avoided during maximum distortion energy criterion, according to von Mises criterion theory. A three-dimensional simulation obtained by FEM search to evaluate tension levels that rise in the archwire body after activation. ML archwire should work in elastic range, i.e., should not surpasses the YS after pre-activation and after activation. This study was restricted to consider only the four anterior brackets due to the degree of stress-strain on the loops. Four blocks simulated the incisors brackets keeping the same interbracket distance. Table 1 shows the material mechanical properties.

**Table 1:** Wire material mechanical properties.

Modulus of elasticity (E)	69 GPa
Yield stress (σe)	1240 MPa
Poisson’s ratio	0.3
Bulk modulus	57.5 GPa
Shear modulus	26.54 GPa

**Figure 3:**
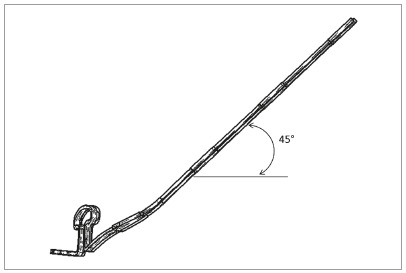
Mushroom archwire and gable bend. At left of the loop, detail of the lateral incisor bracket. At right side, distal extremity, with a gable bend of 45 degrees.

**Figure 4:**
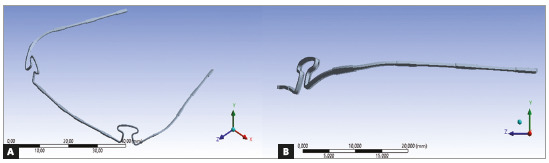
A) Isometric view. B) Detail in anterior view with brackets.

A large displacement for non-linear analysis was used for simulation. The archwires were deformed in their extremities with 0° and 45°(Fig 3) and the loop was activated. For activation, the vertical extremities of the loop were separated at 4.0 or at 5.0 mm. Initially, only pre-activation was considered (vertical extremities separated for 2.5mm, anterior torque and gable bends inserted). Figure 4 shows the archwire activated. FEM performed a convergence analysis of maximum tensions controlling the maximum element size, assigning different sizes 0.60, 0.55, 0.50 and 0.45 mm type tetrahedral 3D quadratic.

## RESULTS

**Figure 5:**
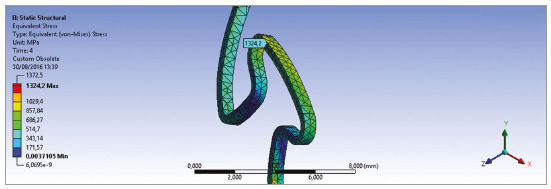
Configuration at maximum tensions.

Table 2 shows the maximum tension values for each element number and the maximum element size. As maximum tensions increase significantly in mesh 3 and decreases in mesh 4, it was observed a convergence in mesh 3 that was adopted in this model. Figure 5 shows the detail of the region where the convergence study was done. It is important to emphasize that although the tension reaches the peak of 1324 MPa that surpass the material’s YS, this value found at 5.0 mm of activation is situated at the top of the loop in a very small punctual area (Fig 5) while a more accurate analysis of the different resulted tensions revealed a maximum tension of 1158 MPa at the whole part of the loop. The von Mises stress analysis revealed that the maximum tensions are not significant for 4.0mm and 5.0mm of activation.

**Table 2:** Convergence study.

Mesh	Maximum face size (mm)	Number of elements	Number of nodes	Maximum Stress (MPa)
1	0.60	4258	9771	1221.10
2	0.55	5399	12230	1220.10
3	0.50	6455	14116	1324.20
4	0.45	8197	17410	1203.80

## DISCUSSION

The present paper evaluated the von Mises stress over a 3-D Mushroom prototype using AutoDesk Inventor software considering the von Mises criterion theory. 

A three-dimensional simulation obtained by FEM was performed. ML archwires with CNA Beta III wire material (0.017 x 0.025-in) are very important appliances to retract anterior teeth with torque and anchorage control.^7^ ^,^ ^9^ ^-^ ^12^ Closing loops incorporated in archwires helps to control anterior torque over incisors during retraction, but they should work in an elastic range to develop a desired force system. Besides spring geometry, properties as low load deflection rates, adequate spring gradient (spring rate) and working range should be considered in closing loops.^13^ ^-^ ^21^ Also, torque control and gable bends are important second-order bends to avoid incisors to move lingually during retraction and posterior anchorage loss, respectively. Retraction closing loops should have a low load/deflection rate and work in an elastic, range to prevent plastic deformation. If the material enters in the plastic regime (surpass YS), the retraction spring cannot develop the minimum load to produce teeth movement.^13^ In this research, ML archwire was evaluated by FEM to know the von Mises stresses resulted from pre-activation and activations. A titanium-molybdenum material with a Yield Strength (YS) of 1240 MPa and a Modulus of Elasticity (E) of 69 GPa was used. It was verified in the present study that the ML archwires are capable to produce safe tensions along the spring body and could be activated up to 5.0 mm without risk of loop deformation. 

In a FEM study^13^ focusing on verifying the von Mises stresses in Delta retraction springs (DRS, TMA, 0.016 x 0.022-in) using numerical and experimental methods, it was concluded that the springs could be activated up to 7.0 mm without surpass the YS. Also, Tear Drop loops were verified^14^ experimentally and numerically concerning the force system and the stress along the loops (SS, 0.019 x 0.025-in). It was found high tension levels at the top of the loop (1201-1352 N/mm^2^). Another paper^15^ verified the behavior of DRS to know the von Mises stresses comparing prototypes with and without helicoids inserted on the top of the springs. The authors concluded that the insertion of helicoids decreases the deflection rates according to early studies.^5^ ^,^ ^20^ ^-^ ^22^ In the present paper, even though the YS obtained (1324 MPa) surpass the material YS (1240 MPa), this value does not represent a significant tension because plastic deformation occurs only at a very small localized point (upper part of the loop). A more accurate analysis of the different tensions obtained over the spring revealed that the maximum tension is about 1158 MPa also at the top of the loop at 5.0 mm of activation. Studies concerning the von Mises tensions show that the higher tensions normally occurs at horizontal legs near the attachments (brackets or molar tubes) and in the superior part of the loops due to energy concentration.^13^ ^,^ ^15^ ^,^ ^16^ ML archwires are a modified version originated from T-loops developed by Nanda^23^ aiming to obtain more flexibility during the controlled retraction or translation of the four incisors, after the canine retraction (two-step procedure).^7^ For an ideal tooth movement of the anterior teeth, the translation movement should have a M:F ratio of approximately 10:1.^7^ ^,^ ^23^

In the present study, a titanium-molybdenum material was considered with the same mechanical properties of TMA wires. The prototypes studied, as well as the Mushroom arches, had their upper portion rounded and were pre-activated at their vertical extremities, spaced 2.5 mm apart. Vertical extremities could be activated from 4.0 mm up to 5.0 mm to produce effective anterior torque to prevent incisors to tip lingually during retraction. Also, incorporated gable bends of 45 degrees in their extremities are made to avoid anchorage loss in the posterior segment. Titanium molybdenum with a nominal composition of 79% Ti, 11% Mo, 6% Zr and 4% Sn has been used clinically. CNA Beta III alloys are nickel-free, and prevents allergies in some patients, have a good range and are about 42% less stiff than stainless steel.^23^ Many papers show case reports^8^ ^-^ ^12^ dealing with CNA Beta III alloys, but no study demonstrates experimentally or numerically what occurs in the loop body with Mushroom geometry neither their force system after activation. In simulations, the results represent the behavior of the same object that is based on its theoretical model, so there is no variation in the material behavior. On the other hand, in the experimental method, the real behavior of an object and error must be verified statistically in order to certify that this error lies within certain limits. Further experimental studies are necessary to obtain the moments (Mx, My and Mz), forces (Fx, Fy and Fz) and the M/F ratios.

## CONCLUSIONS

» Mushroom loop 0.017 x 0.025-in archwires are capable to produce tension levels in an elastic range and could be activated safely up to 5.0mm.
» Tensions revealed a maximum of 1158 MPa at the whole part of the loop at 5.0mm of activation, except in a very small area situated at the top of the loop, in which a maximum of 1324 MPa was found.
